# Supplementary material for: A Likelihood Approach for Real-Time Calibration of Stochastic Compartmental Epidemic Models
Source: PLoS Comput Biol. 2017 Jan 17;13(1):e1005257. doi: 10.1371/journal.pcbi.1005257 (PMC5240920; doi:10.1371/journal.pcbi.1005257)
Supplement: S1 File — (TAR.GZ) [file pcbi.1005257.s014.tar.gz › HSPH_Online-SI-Revision/IPoi09/n10-severe/n10-severe_BENCH_table0.pdf]

1	R0	{1.68952, 11.8402}	{2.64373, 19.7855}	{{2.64373, 19.7855}, {2.64373, 19.7855}}	{56.4782, 67.1044}	inf	1046.	{1488., 2441.}	90.2546	0.271853	newly	589.	670.54	{491., 856.}	22.4728	0.0885562	Reff	0.898487	0.761675	{0.440181, 1.28538}	41.4169	ar	6872.	9088.49	{9032., 9142.}	32.254
2	R0	{1.71739, 9.62661}	{2.6224, 18.4517}	{{2.59755, 18.2464}, {2.76795, 19.6041}}	{52.6966, 91.6736}	inf	945.	2124.18	{1675., 2563.}	134.26	0.3857	newly	711.	789.419	{605., 961.}	25.7157	0.129806	Reff	0.984408	1.06681	{0.564662, 1.8253}	41.0084	ar	6874.	9080.87	{8990., 9231.}	32.1046
3	R0	{1.48936, 6.23975}	{2.93488, 18.8445}	{{2.93488, 18.8445}, {2.93488, 18.8445}}	{97.0565, 202.007}	inf	625.	2486.97	{1227., 2937.}	300.391	0.578173	newly	640.	908.656	{626., 1085.}	52.5666	0.196031	Reff	0.888701	1.12714	{0.559095, 2.33558}	47.3851	ar	5666.	9360.51	{9324., 9401.}	65.2049
4	R0	{1.62394, 7.51135}	{2.42205, 19.4475}	{{2.42205, 19.4475}, {2.42205, 19.4475}}	{49.1465, 158.908}	inf	753.	1754.26	{1131., 2182.}	139.509	0.391093	newly	670.	611.155	{372., 773.}	20.2342	0.124647	Reff	0.945133	0.90679	{0.493856, 1.48738}	38.1841	ar	6417.	8811.98	{8743., 8883.}	37.3225
5	R0	{1.53632, 8.57652}	{2.42205, 19.4475}	{{2.42205, 19.4475}, {2.42205, 19.4475}}	{57.6527, 126.753}	inf	707.	1754.26	{1131., 2182.}	154.419	0.415636	newly	572.	611.155	{372., 773.}	25.5154	0.132165	Reff	0.929627	0.90679	{0.493856, 1.48738}	38.3609	ar	6260.	8811.98	{8743., 8883.}	40.7665
6	R0	{1.47166, 5.7434}	{2.42205, 19.4475}	{{2.42205, 19.4475}, {2.42205, 19.4475}}	{64.5795, 238.606}	inf	531.	1787.69	{1102., 2212.}	245.47	0.556622	newly	615.	647.776	{468., 799.}	22.9465	0.14921	Reff	0.960847	1.21201	{0.671634, 1.93352}	44.6771	ar	5650.	8811.98	{8743., 8883.}	55.9643
7	R0	{1.48179, 8.87369}	{2.35657, 16.9254}	{{2.35866, 16.9062}, {2.35866, 16.9062}}	{59.0356, 90.7372}	inf	480.	1574.27	{969., 2032.}	230.311	0.509433	newly	360.	609.136	{379., 815.}	73.0367	0.234297	Reff	0.902706	0.735084	{0.457108, 1.11541}	37.0574	ar	5233.	8715.49	{8629., 8794.}	66.5487
8	R0	{1.53669, 6.47807}	{2.87021, 19.0518}	{{2.86656, 18.9813}, {2.86656, 18.9813}}	{86.7786, 194.097}	inf	675.	2454.7	{1918., 2897.}	264.118	0.552975	newly	686.	880.685	{690., 1046.}	31.395	0.117273	Reff	0.902344	0.960315	{0.494768, 1.77096}	39.9072	ar	5934.	9314.55	{9264., 9378.}	56.9691
9	R0	{1.57227, 6.20989}	{2.96827, 15.9544}	{{2.96947, 15.963}, {2.96947, 15.963}}	{88.7886, 156.918}	inf	741.	2527.32	{1488., 3029.}	247.949	0.539254	newly	787.	1124.16	{843., 1319.}	54.059	0.213251	Reff	1.05279	1.28258	{0.693074, 2.26571}	43.226	ar	6136.	9382.29	{9318., 9450.}	52.9056
10	R0	{1.54936, 6.50938}	{2.79693, 12.6075}	{{2.75598, 12.4651}, {2.85273, 12.8842}}	{80.5214, 93.6813}	inf	717.	2203.86	{1485., 2748.}	207.373	0.475041	newly	740.	1130.37	{748., 1452.}	54.5298	0.179069	Reff	0.879107	0.778908	{0.411364, 1.2683}	41.1019	ar	6275.	9243.51	{9178., 9319.}	47.3069
11	R0	{1.42179, 3.18601}	{2.7499, 15.3124}	{{2.7499, 15.3124}, {2.7499, 15.3124}}	{93.4111, 380.614}	inf	449.	2280.32	{1564., 2713.}	408.514	0.683483	newly	893.	1051.18	{845., 1221.}	26.715	0.114091	Reff	0.921746	1.2287	{0.645402, 1.98845}	48.0238	ar	5322.	9203.37	{9154., 9249.}	72.9307
12	R0	{1.48035, 6.46183}	{2.86977, 16.8843}	{{2.73108, 15.6124}, {2.96398, 17.7348}}	{93.8573, 161.294}	inf	718.	2431.56	{1921., 2917.}	242.369	0.528592	newly	750.	1031.62	{944., 1199.}	45.4329	0.17865	Reff	0.99065	1.34491	{0.736845, 1.91355}	47.4446	ar	5793.	9314.66	{9213., 9402.}	60.7917
13	R0	{1.75755, 8.53854}	{2.8596, 19.9027}	{{2.85956, 19.8999}, {2.85956, 19.8999}}	{62.7039, 133.092}	inf	1074.	2389.7	{1971., 2782.}	126.611	0.356633	newly	871.	812.443	{620., 991.}	14.7936	0.0795188	Reff	0.980361	0.901871	{0.482694, 1.40519}	41.1352	ar	6832.	9301.31	{9253., 9343.}	36.1433
14	R0	{1.47355, 4.89451}	{2.75142, 15.3401}	{{2.7499, 15.3124}, {2.7499, 15.3124}}	{86.7205, 213.414}	inf	528.	2286.15	{1773., 2665.}	332.982	0.625508	newly	648.	1003.35	{769., 1207.}	56.4217	0.188751	Reff	0.862321	0.938471	{0.492782, 1.57954}	41.1873	ar	5710.	9205.53	{9154., 9253.}	61.2177
15	R0	{1.62071, 5.98665}	{2.60697, 9.44648}	{{2.59708, 9.39342}, {2.59708, 9.39342}}	{60.8534, 57.7925}	inf	906.	2011.49	{1456., 2535.}	123.646	0.340738	newly	967.	1340.18	{836., 1785.}	43.4349	0.150774	Reff	0.893659	0.791797	{0.44462, 1.28815}	38.5793	ar	6635.	9066.82	{8991., 9144.}	36.6513
16	R0	{1.67268, 7.7256}	{2.97925, 15.85}	{{2.96947, 15.6207}, {2.99911, 15.963}}	{78.1124, 105.163}	inf	927.	1873.53	{1205., 2453.}	102.107	0.288455	newly	767.	739.984	{460., 1037.}	24.7494	0.112936	Reff	0.766087	0.494137	{0.293613, 0.694894}	52.542	ar	6947.	9398.41	{9346., 9454.}	35.2873
17	R0	{1.67805, 11.1417}	{2.50578, 14.5234}	{{2.36011, 13.1698}, {2.55942, 15.0551}}	{49.3267, 30.3518}	inf	929.	1953.32	{1271., 2404.}	116.03	0.337689	newly	581.	894.153	{539., 1138.}	61.5129	0.214517	Reff	1.07479	0.882035	{0.475796, 1.42585}	36.1837	ar	6625.	8925.1	{8732., 9069.}	34.7185
18	R0	{1.66743, 9.99046}	{2.84557, 19.7352}	{{2.85956, 19.8999}, {2.85956, 19.8999}}	{70.6561, 97.5404}	inf	904.	2409.52	{1795., 2791.}	172.546	0.439694	newly	639.	844.23	{689., 984.}	40.4141	0.165463	Reff	0.998457	1.13168	{0.594503, 1.79323}	42.5753	ar	6899.	9286.9	{9241., 9343.}	34.6123
19	R0	{1.70787, 9.85478}	{2.46477, 18.9032}	{{2.46477, 18.9032}, {2.46477, 18.9032}}	{44.3184, 91.8176}	inf	940.	1864.48	{1335., 2246.}	103.378	0.320832	newly	617.	666.1	{443., 824.}	22.0421	0.112023	Reff	0.877333	0.897567	{0.478658, 1.46876}	37.2637	ar	6867.	8883.57	{8780., 8947.}	29.3661
20	R0	{1.53809, 10.5848}	{2.29144, 19.4322}	{{2.23915, 18.4504}, {2.30371, 19.6625}}	{48.9798, 83.5859}	inf	660.	1627.14	{963., 2008.}	152.223	0.407105	newly	432.	590.641	{389., 737.}	46.488	0.185347	Reff	1.05928	1.10296	{0.629604, 1.83997}	36.0512	ar	6108.	8600.31	{8475., 8703.}	40.8041
21	R0	{1.59925, 6.74479}	{2.89549, 13.7166}	{{2.71225, 12.7047}, {2.95209, 14.037}}	{81.0528, 103.366}	inf	837.	2437.07	{1769., 2962.}	191.167	0.454457	newly	789.	1172.74	{848., 1447.}	49.2404	0.166622	Reff	0.912212	0.881979	{0.457574, 1.63192}	40.9466	ar	6411.	9329.23	{9225., 9409.}	45.5192
22	R0	{1.38779, 4.40977}	{2.16962, 10.9957}	{{2.14747, 10.7247}, {2.31035, 12.8066}}	{56.3362, 149.348}	inf	333.	1532.79	{1221., 1879.}	360.298	0.649977	newly	507.	923.094	{697., 1128.}	83.0151	0.254688	Reff	0.993103	0.885235	{0.583897, 1.36364}	29.229	ar	5154.	8387.17	{8268., 8673.}	62.7313
23	R0	{1.53657, 7.50352}	{2.32188, 12.5902}	{{2.2885, 12.1239}, {2.42221, 13.5694}}	{51.1079, 67.7905}	inf	535.	1434.22	{769., 2007.}	168.262	0.406167	newly	479.	729.523	{371., 1061.}	59.6885	0.195302	Reff	0.991395	0.667118	{0.419711, 1.00877}	40.6424	ar	5928.	8668.43	{8572., 8784.}	46.2286
24	R0	{1.71131, 6.24526}	{2.93385, 18.8113}	{{2.93488, 18.8445}, {2.93488, 18.8445}}	{71.4387, 201.209}	inf	964.	2379.24	{719., 2948.}	160.65	0.432834	newly	1016.	910.565	{380., 1100.}	16.2132	0.119261	Reff	0.982634	1.41941	{0.738709, 2.59619}	54.7181	ar	6924.	9360.11	{9324., 9401.}	35.1836
25	R0	{1.4412, 3.07729}	{2.96307, 14.3219}	{{2.96307, 14.3219}, {2.96307, 14.3219}}	{105.597, 365.406}	inf	606.	2533.94	{1277., 3012.}	322.581	0.609395	newly	1165.	1254.27	{871., 1461.}	20.7684	0.105064	Reff	0.935195	1.28561	{0.691284, 2.3432}	50.1436	ar	5810.	9382.58	{9331., 9427.}	61.4902
26	R0	{1.4159, 4.93858}	{2.10405, 8.58066}	{{2.10405, 8.58066}, {2.10405, 8.58066}}	{48.6016, 73.7475}	inf	458.	1381.59	{951., 1639.}	201.657	0.469526	newly	608.	1055.04	{639., 1346.}	75.8675	0.235539	Reff	0.962104	0.863263	{0.537374, 1.28747}	28.9125	ar	5255.	8220.37	{8131., 8343.}	56.4295
27	R0	{1.57465, 7.05414}	{2.72375, 12.7631}	{{2.71225, 12.7047}, {2.71225, 12.7047}}	{72.9752, 80.9305}	inf	797.	2223.06	{1261., 2639.}	185.174	0.451311	newly	776.	1234.92	{936., 1472.}	66.4478	0.228829	Reff	1.05486	1.24474	{0.62436, 2.06782}	39.7148	ar	6359.	9172.23	{9101., 9239.}	44.2401
28	R0	{1.41422, 4.62402}	{2.96947, 15.963}	{{2.96947, 15.963}, {2.96947, 15.963}}	{109.972, 245.219}	inf	570.	2525.31	{1488., 3029.}	348.605	0.639795	newly	804.	1123.48	{843., 1319.}	51.304	0.206452	Reff	0.944699	1.29323	{0.693074, 2.26571}	50.0181	ar	5485.	9383.39	{9320., 9450.}	71.0736
29	R0	{1.41901, 3.63017}	{2.87644, 11.295}	{{2.70795, 10.355}, {2.94803, 11.6547}}	{102.708, 211.142}	inf	600.	2496.26	{2037., 2920.}	316.044	0.609303	newly	1020.	1455.93	{1157., 1738.}	45.1302	0.158071	Reff	0.854528	0.888814	{0.51996, 1.3849}	33.263	ar	5979.	9296.3	{9130., 9389.}	55.4824
30	R0	{1.60719, 4.72984}	{2.98417, 12.7347}	{{2.96796, 12.4643}, {2.9884, 12.8326}}	{85.6765, 169.241}	inf	850.	2588.84	{1583., 3020.}	206.863	0.479	newly	1189.	1408.11	{1111., 1637.}	24.7368	0.101796	Reff	0.978457	1.17115	{0.582738, 2.19169}	44.9076	ar	6479.	9401.96	{9341., 9455.}	45.1144
31	R0	{1.38175, 6.26468}	{2.10405, 8.58066}	{{2.10405, 8.58066}, {2.10405, 8.58066}}	{52.2743, 36.9688}	inf	497.	1183.9	{624., 1688.}	138.509	0.352866	newly	506.	851.039	{400., 1294.}	76.8697	0.235176	Reff	0.889156	0.682871	{0.477619, 0.968284}	30.6625	ar	5397.	8220.37	{8131., 8343.}	52.3137
32	R0	{1.4947, 8.8847}	{2.24467, 16.0082}	{{2.24242, 15.7579}, {2.24606, 16.668}}	{50.1752, 80.1773}	inf	437.	1373.72	{720., 1920.}	215.621	0.474426	newly	341.	563.033	{303., 820.}	71.4196	0.220998	Reff	0.948387	0.733512	{0.434805, 1.03907}	36.5133	ar	5407.	8528.21	{8419., 8637.}	57.7254
33	R0	{1.55891, 8.28978}	{2.42205, 19.4475}	{{2.42205, 19.4475}, {2.42205, 19.4475}}	{55.3682, 134.596}	inf	655.	1754.26	{1131., 2182.}	173.796	0.445382	newly	521.	611.155	{372., 773.}	31.6202	0.14714	Reff	0.96668	0.90679	{0.493856, 1.48738}	37.9931	ar	6239.	8811.98	{8743., 8883.}	41.2403
34	R0	{1.60764, 10.4729}	{2.46477, 18.9032}	{{2.46477, 18.9032}, {2.46477, 18.9032}}	{53.316, 80.4963}	inf	772.	1864.48	{1335., 2246.}	145.702	0.398805	newly	469.	666.1	{443., 824.}	48.408	0.18649	Reff	0.879861	0.897567	{0.478658, 1.46876}	37.2244	ar	6495.	8883.57	{8780., 8947.}	36.7755
35	R0	{1.48527, 5.74485}	{2.7499, 15.3124}	{{2.7499, 15.3124}, {2.7499, 15.3124}}	{85.1448, 166.541}	inf	650.	2288.1	{1773., 2665.}	252.015	0.535633	newly	769.	1005.15	{769., 1207.}	34.3995	0.126252	Reff	0.956217	0.937499	{0.492782, 1.57954}	39.1586	ar	5811.	9203.37	{9154., 9249.}	58.3784
36	R0	{1.61457, 9.93364}	{2.64199, 19.7627}	{{2.64373, 19.7855}, {2.64373, 19.7855}}	{63.6343, 98.9472}	inf	799.	2151.69	{1504., 2559.}	171.945	0.424743	newly	557.	759.281	{595., 893.}	43.1946	0.162729	Reff	1.02993	1.101	{0.584264, 1.89608}	38.6441	ar	6338.	9084.41	{9020., 9142.}	43.3324
37	R0	{1.47831, 7.08139}	{2.30984, 12.1198}	{{2.27199, 11.6669}, {2.52651, 14.1724}}	{56.2486, 71.1494}	inf	564.	1638.09	{937., 2194.}	199.18	0.486508	newly	542.	894.125	{542., 1159.}	74.5882	0.26113	Reff	0.993129	1.01787	{0.540279, 2.06794}	37.8105	ar	5530.	8665.98	{8460., 9052.}	56.7085
38	R0	{1.73445, 9.8858}	{2.88709, 17.2325}	{{2.8833, 17.1847}, {2.98558, 18.1089}}	{66.4557, 74.3155}	inf	986.	2493.78	{1833., 2894.}	157.4	0.410083	newly	672.	998.766	{740., 1169.}	54.1922	0.193351	Reff	1.0313	1.08326	{0.563108, 1.77546}	35.6699	ar	6842.	9317.05	{9236., 9391.}	36.1743
39	R0	{1.66621, 11.4824}	{2.63677, 19.6943}	{{2.64373, 19.7855}, {2.64373, 19.7855}}	{58.2496, 71.5173}	inf	863.	2143.02	{1504., 2533.}	151.499	0.392384	newly	517.	757.081	{595., 893.}	52.7516	0.189276	Reff	1.00256	1.10724	{0.584264, 1.89608}	39.698	ar	6473.	9083.66	{9020., 9136.}	40.3316
40	R0	{1.60694, 9.95741}	{2.64373, 19.7855}	{{2.64373, 19.7855}, {2.64373, 19.7855}}	{64.5195, 98.7013}	inf	852.	2114.49	{1722., 2533.}	149.216	0.389119	newly	560.	727.54	{567., 887.}	33.1548	0.122718	Reff	0.960147	0.920039	{0.51024, 1.59576}	39.8178	ar	6625.	9088.49	{9032., 9142.}	37.1848
41	R0	{1.55128, 4.71911}	{2.76852, 11.3068}	{{2.52701, 9.79002}, {2.99519, 12.8326}}	{78.4671, 139.596}	inf	710.	2351.48	{1732., 2981.}	232.813	0.512322	newly	1008.	1409.14	{1086., 1647.}	43.0039	0.155673	Reff	1.00632	1.0799	{0.595743, 1.82652}	38.1525	ar	6141.	9199.21	{8913., 9449.}	49.7999
42	R0	{1.60063, 6.81118}	{2.4379, 19.0649}	{{2.43839, 19.0531}, {2.43839, 19.0531}}	{52.3088, 179.906}	inf	843.	1806.79	{1344., 2217.}	127.279	0.391964	newly	835.	661.672	{521., 811.}	20.8404	0.152814	Reff	0.933487	1.15709	{0.611061, 1.80343}	44.4497	ar	6528.	8838.71	{8751., 8912.}	35.3969
43	R0	{1.56986, 5.44652}	{2.82209, 12.049}	{{2.8186, 12.0293}, {2.8186, 12.0293}}	{79.7672, 121.224}	inf	706.	2325.55	{1647., 2792.}	230.172	0.506856	newly	846.	1250.13	{875., 1531.}	50.3861	0.172584	Reff	0.905652	0.855692	{0.4273, 1.59138}	39.91	ar	6312.	9279.45	{9219., 9337.}	47.0128
44	R0	{1.47523, 8.07785}	{2.59381, 18.207}	{{2.59755, 18.2464}, {2.59755, 18.2464}}	{75.824, 125.394}	inf	601.	2068.13	{1269., 2518.}	251.649	0.545026	newly	507.	781.787	{564., 971.}	64.4615	0.235541	Reff	0.965243	1.0721	{0.581591, 2.017}	42.0483	ar	5692.	9047.8	{8982., 9121.}	58.9565
45	R0	{1.6417, 7.69429}	{2.7499, 15.3124}	{{2.7499, 15.3124}, {2.7499, 15.3124}}	{67.5032, 99.0099}	inf	911.	2288.1	{1773., 2665.}	151.818	0.392208	newly	777.	1005.15	{769., 1207.}	33.2565	0.122809	Reff	0.893577	0.937499	{0.492782, 1.57954}	39.9506	ar	6735.	9203.37	{9154., 9249.}	36.6499
46	R0	{1.38865, 5.83473}	{1.93462, 9.38215}	{{1.93462, 9.38215}, {1.93462, 9.38215}}	{39.3166, 60.7984}	inf	383.	1076.75	{665., 1313.}	181.136	0.437588	newly	442.	728.025	{416., 932.}	68.5351	0.218829	Reff	0.981081	0.749854	{0.566457, 1.00445}	28.396	ar	5233.	7776.53	{7616., 7920.}	48.6055
47	R0	{1.45289, 6.87903}	{1.93419, 8.56054}	{{1.93419, 8.56054}, {1.93419, 8.56054}}	{33.1271, 24.444}	inf	477.	1000.05	{681., 1356.}	111.116	0.30951	newly	487.	719.098	{456., 1041.}	53.5383	0.176899	Reff	1.01252	0.692398	{0.553178, 0.904234}	33.9368	ar	5379.	7790.85	{7670., 7956.}	44.8383
48	R0	{1.69416, 9.4831}	{2.64373, 19.7855}	{{2.64373, 19.7855}, {2.64373, 19.7855}}	{56.0496, 108.64}	inf	944.	2085.49	{1045., 2552.}	129.617	0.36277	newly	702.	751.524	{452., 913.}	24.4607	0.123412	Reff	1.03412	1.32781	{0.734428, 2.13798}	41.855	ar	6925.	9088.49	{9032., 9142.}	31.2418
49	R0	{1.47843, 7.16286}	{2.54276, 15.4314}	{{2.45256, 14.4765}, {2.65407, 16.6623}}	{71.9904, 115.437}	inf	577.	2066.01	{1511., 2487.}	259.492	0.547787	newly	548.	907.366	{679., 1068.}	67.3923	0.221317	Reff	0.972659	0.992472	{0.551065, 1.76882}	34.5387	ar	5449.	8970.01	{8837., 9137.}	64.6176
50	R0	{1.68843, 5.72427}	{2.84073, 10.0565}	{{2.84073, 10.0565}, {2.84073, 10.0565}}	{68.2468, 75.6818}	inf	853.	2105.8	{1475., 2717.}	146.87	0.382314	newly	913.	1278.13	{827., 1800.}	43.0705	0.146517	Reff	0.853333	0.641456	{0.366454, 0.924374}	43.0753	ar	6800.	9282.63	{9214., 9332.}	45.5093
